# Ground-State Structures of Hydrated Calcium Ion Clusters From Comprehensive Genetic Algorithm Search

**DOI:** 10.3389/fchem.2021.637750

**Published:** 2021-06-30

**Authors:** Ruili Shi, Zhi Zhao, Xiaoming Huang, Pengju Wang, Yan Su, Linwei Sai, Xiaoqing Liang, Haiyan Han, Jijun Zhao

**Affiliations:** ^1^ School of Mathematics and Physics, Hebei University of Engineering, Handan, China; ^2^ Key Laboratory of Materials Modification by Laser, Ion and Electron Beams, Ministry of Education, Dalian University of Technology, Dalian, China; ^3^ School of Ocean Science and Technology, Dalian University of Technology, Panjin Campus, Panjin, China; ^4^ Department of Mathematics and Physics, Hohai University, Changzhou, China; ^5^ School of Electronics and Information Engineering, Taizhou University, Taizhou, China

**Keywords:** hydrated calcium ion cluster, genetic algorithm, hydrogen bond, coordination number, natural bond orbital

## Abstract

We searched the lowest-energy structures of hydrated calcium ion clusters Ca^2+^(H_2_O)_n_ (*n* = 10–18) in the whole potential energy surface by the comprehensive genetic algorithm (CGA). The lowest-energy structures of Ca^2+^(H_2_O)_10–12_ clusters show that Ca^2+^ is always surrounded by six H_2_O molecules in the first shell. The number of first-shell water molecules changes from six to eight at *n* = 12. In the range of *n* = 12–18, the number of first-shell water molecules fluctuates between seven and eight, meaning that the cluster could pack the water molecules in the outer shell even though the inner shell is not full. Meanwhile, the number of water molecules in the second shell and the total hydrogen bonds increase with an increase in the cluster size. The distance between Ca^2+^ and the adjacent water molecules increases, while the average adjacent O-O distance decreases as the cluster size increases, indicating that the interaction between Ca^2+^ and the adjacent water molecules becomes weaker and the interaction between water molecules becomes stronger. The interaction energy and natural bond orbital results show that the interaction between Ca^2+^ and the water molecules is mainly derived from the interaction between Ca^2+^ and the adjacent water molecules. The charge transfer from the lone pair electron orbital of adjacent oxygen atoms to the empty orbital of Ca^2+^ plays a leading role in the interaction between Ca^2+^ and water molecules.

## Introduction

It is well acknowledged that most of the biochemistry reactions accomplished by ions happen in the water environment. The activation of water by metal ions and charge transfer to solvent originating from a metal ion are of fundamental importance for understanding the hydrogen bond formation in aqueous environments (Kistenmacher et al., 1974; Rudolph and Irmer, 2013; Chen and Ruckenstein, 2015; Hadad et al., 2019). By the end of the last century, a series of works used molecular dynamics (MD) or density functional theory (DFT) to study the hydrated ion clusters. These research focused on the structures, the coordination number (CN) of the metal ion, the interaction of ion–solvent or solvent–solvent, and the influential factors of hydration bond structures (Caldwell et al., 1990; Hall et al., 2000; Hofer et al., 2005; Fujiwara et al., 2010; Boda et al., 2012; Yoo et al., 2016; Delgado et al., 2020). For example, Fujiwara et al. used the fragment molecular orbital–based molecular dynamics (FMO-MD) method to investigate the hydration structure of the droplet containing a divalent zinc ion and 64 water molecules and provided the CN of 6 (Fujiwara et al., 2010). Hofer et al. made the comparison of *ab initio* quantum mechanical/molecular mechanical (QM/MM) molecular dynamics (MD) simulations with those of classical simulations based on the pair potential added by three-body interaction potentials to accentuate the difference of the “quantum effect” in the hydrated Ba (II) ion (Hofer et al., 2005). The investigation from microhydration to bulk hydration of the Sr^2+^ ion has been accomplished by Anil Boda et al. using DFT, MP2, and the molecular dynamics study (Boda et al., 2012). The experimental studies could yield considerable results and make the structural and physical properties obtained from theoretical studies more credible (Misaizu et al., 1995; Siu et al., 2002; Buck et al., 2007; Gao and Liu, 2007; Carrera et al., 2009; Zhang and Liu, 2011). All these make us understand much deeper the actual reactions outside the laboratory and inside organisms.

Calcium, as one of the most important ions in the tissue fluid, participates in many biochemical reactions such as exocytosis, neurotransmitter release, and many vital movements such as muscle contraction or electrical conduction of the heart (Prendergast and Mann, 1977; Hewish et al., 1982; Chizhik et al., 2016). The fundamental study is to describe the CN and microstructures of the hydrated calcium ion clusters since the biological or chemical properties are determined by the structures of hydrated calcium ion clusters (Bakó et al., 2002; González et al., 2005).

In experiments, the CN of the calcium ion varies largely from 5 to 10 by using X-ray diffraction, neutron diffraction, extended X-ray absorption fine structure spectroscopy, and other techniques (Hewish et al., 1982; Probst et al., 1985; Marcus, 1988; Yamaguchi et al., 1989; Peschke et al., 1998; Jalilehvand et al., 2001; Fulton et al., 2003; Megyes et al., 2004). It should be ascribed to the different conditions and environments of water molecules. The ratio between water molecules and ions also could change the CN. For example, when the ion concentration is smaller, the number of first-shell water molecules will be larger (Hewish et al., 1982; Yamaguchi et al., 1989; Jalilehvand et al., 2001; Megyes et al., 2004). Moreover, infrared (IR) spectra could help provide the evolution information of the structures of the hydrated calcium ion clusters (Butler et al., 2014). Williams and colleagues conducted a series of IR spectroscopy of Ca^2+^(H_2_O)_n_ with *n* = 4–69 in experiments (Bush et al., 2007; Bush et al., 2008; Bush et al., 2009). Their results revealed that there are six water molecules adjacent to the calcium dication for Ca^2+^(H_2_O)_n_ with *n* = 6–10 clusters (Bush et al., 2007), and the number of water molecules in the first hydration shell changes from six to eight at *n* ≈ 12 (Bush et al., 2008). Recently, the binding energy of hydrated calcium ion clusters with up to *n* = 20 was measured by Bruzzi and Stace using the pick-up technique in conjunction with finite heat bath theory to characterize the interaction between calcium ions with the multi-outer shell water molecules (Bruzzi and Stace, 2017). Their results showed that there are six water molecules in the first hydration shell, and the 2+ charge on the calcium cation has an influence on the molecular interactions that extends far beyond the first hydration shell.

Meanwhile, the CN of the calcium ion and the structures of the hydrated calcium ion attracted lots of attention on the theoretical side. Using a semiempirical coupling method with a basin-hopping global optimization approach, Wales and co-workers searched the low-lying structures of Ca^2+^(H_2_O)_n_ with *n* = 1–20 clusters, showing that Ca^2+^ prefers to locate at the center of the cluster surrounded by eight adjacent water molecules (González et al., 2005). The CN of the calcium ion attained by Monte Carlo (MC) (Bernal‐Uruchurtu and Ortega‐Blake, 1995) and MD (Obst and Bradaczek, 1996; Tongraar et al., 1997; Todorova et al., 2008; Wanprakhon et al., 2011; León-Pimentel et al., 2018) studies varies from 6 to 10. However, the results from DFT calculations showed that there are six water molecules in the first hydration shell (Megyes et al., 2004; Peschke et al., 2000; Carl et al., 2007; Lei and Pan, 2010). For example, the work by Lei and Pan at the BLYP/6-311+G(d,p) level of theory showed that the first and second hydration shells of the lowest-energy structures of Ca^2+^(H_2_O)_n_ with *n* = 1–20 and 27 are fully occupied by six and nine water molecules, respectively (Lei and Pan, 2010). The discrepancy may be caused by the different computational methods. All the studies imply that still there is some controversy about the ground state structures of medium-sized hydrated calcium ion clusters, especially for the influence of the interaction between Ca^2+^ and the second- or even the third-shell water molecules on the number of first-shell water molecules. The evolution process of the first and second hydration shells mainly occurs in the range of *n* = 10–18. Therefore, more efforts are needed to search the potential energy surface (PES) of Ca^2+^(H_2_O)_n_ clusters with *n* = 10–18.

In this work, we use the comprehensive genetic algorithm (CGA) (Zhao et al., 2016) combined with DFT to globally search the PES of hydrated calcium ion clusters Ca^2+^(H_2_O)_n_ with *n* = 10–18. The contrast with the structures given by precedent works and the evolution of the structures with the growth of size is also shown. Finally, we present the interaction between Ca^2+^ and the water molecules using natural bond orbital (NBO) analyses. This work concentrates on the competition between the first- and second-shell water molecules and shows some new low-energy structures of hydrated calcium ion clusters. Our theoretical results provide useful guidance for analyzing the hydrated calcium ion clusters in experiments.

## Method

The CGA (Zhao et al., 2016) has been proved to be outstanding for searching the lowest-energy structures of protonated water clusters and fluoride anion–water clusters (Shi et al., 2017; Shi et al., 2018a; Shi et al., 2018b). We used the CGA combined with the DMol^3^ program (Delley, 1990; Delley, 2000) based on DFT to globally search the PES of Ca^2+^(H_2_O)_n_ with *n* = 10–18. All structures generated by the CGA were fully relaxed with DFT without any symmetry constraint. The double-numerical basis including *p*- and *d*-polarization functions (DNP) and the Becke’s exchange functional (Becke, 1988) and the correlation functional by Lee, Yang, and Parr (BLYP) (Lee et al., 1988) were adopted. The self-consistent field (SCF) density calculations were carried out with a convergence criterion of 10^–6^ a.u. on the total energy.

BLYP and B3LYP (Stephens et al., 1994) as well as 6-311++G(d,p), 6-311+G(d,p), or 6-31+G(d,p) basis set are usually used to describe the hydrated calcium ion clusters (Bakó et al., 2002; Bush et al., 2008; Bush et al., 2009; Lei and Pan, 2010). Meanwhile, MP2 (Møller and Plesset, 1934) is treated as a replacement of CCSD(T) to get more accurate results with low cost for hydrogen bond systems (Shi et al., 2017; Wang et al., 2019; Shi et al., 2020). Thus, we choose MP2 combined with high-level basis set 6-311++G(2d,2p) to evaluate these methods and basis set for describing the geometries of hydrated calcium ion clusters. All the calculations were done with Gaussian 09 package (Frisch et al., 2009). The differences of average adjacent O-O distances and average adjacent O-Ca distances of Ca^2+^(H_2_O)_10_ cluster isomers between several methods and MP2/6-311++G(2d,2p) results are shown in Supplementary Table S1. First, the results of MP2 with 6-311++G(d,p), 6-311+G(d,p), and 6-31+G(d,p) basis set show that the 6-311+G(d,p) basis set could give the most similar results to the 6-311++G(2d,2p) basis set. Thus, we choose 6-311+G(d,p) basis set to optimize hydrated calcium ion clusters. Then the results from BLYP/6-311+G(d,p) and B3LYP/6-311+G(d,p) reveal that B3LYP is outstanding. Finally, we evaluated the dispersion correction on the B3LYP method. We can see from the results of B3LYP/6-311+G(d,p) and B3LYP-D3/6-311+G(d,p) that B3LYP-D3 could give a better geometry of Ca^2+^(H_2_O)_10_ clusters. Considering the computational cost and accuracy, we chose B3LYP-D3/6-311+G(d,p) to optimize the structures of hydrated calcium ion clusters.

Frequency calculations were carried out at the B3LYP-D3/6-311+G(d,p) level of theory, which is same with optimization to ensure each cluster is the true local minimum without imaginary frequency as well as to obtain the zero-point energy (ZPE) and thermal correction at 298 K. Furthermore, the single-point energy (SPE) was calculated at the MP2/6-311++G(2d,2p) level of theory to get more accurate energy. The basis set superposition error (BSSE) correction is considered for the interaction energy. The BSSE correction (ΔE_BSSE_) is based on the site–site function counterpoise method proposed by Wells and Wilson (1983), which is defined as:

$$\Delta E_{BSSE}=\sum_{i=1}^{m}\left[E^{full}\left(fragm\right)-E^{fragm}\left(fragm\right)\right],$$ (1)

where superscript *full* or *fragm* is the energy calculated in the full basis set or in the fragment basis set, and *m* is the number of fragment for a given cluster. Moreover, NBO analyses were performed at the MP2/6-311++G(2d,2p) level of theory based on the B3LYP-D3/6-311+G(d,p) optimization to obtain the charge transfer between calcium ion and water molecules as well as the natural charge of the clusters.

## Results and Discussion

### Lowest-Energy Structures

**FIGURE 1:**
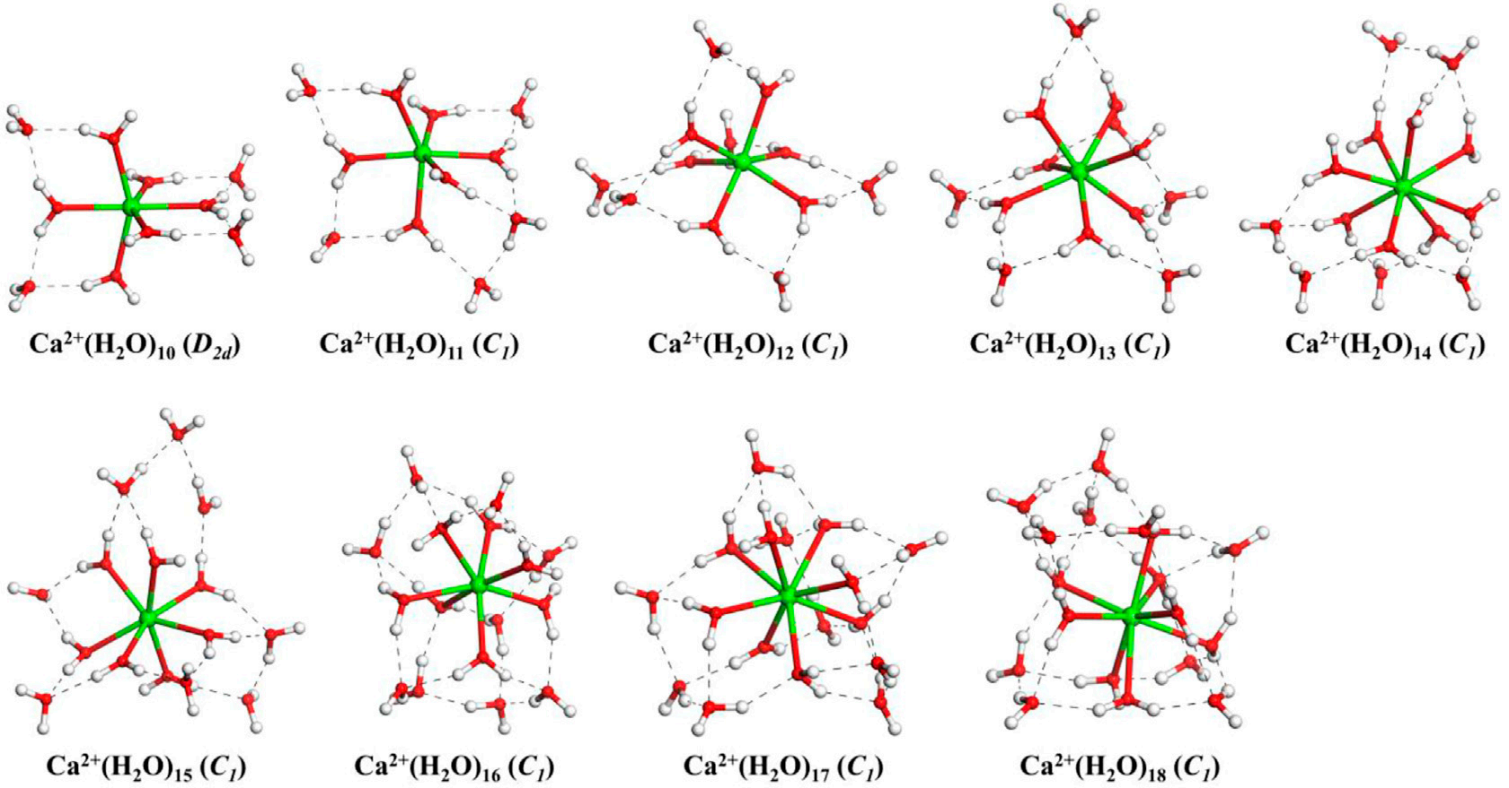
The lowest-energy structures of Ca^2+^(H_2_O)_n_ clusters with *n* = 10–18. The symmetries in parentheses are the symmetries of clusters without hydrogen atoms. The green, red, and white balls denote Ca, O, and H atoms, respectively. The dashed black lines represent the hydrogen bonds.

Figure 1 shows the lowest-energy structures and symmetry of Ca^2+^(H_2_O)_n_ clusters with *n* = 10–18 obtained from the CGA global search. The number of water molecules in the first (N_1_), second (N_2_), and third (N_3_) hydration shells and the number of hydrogen bonds of the lowest-energy structures are listed in Table 1. For comparison, the lowest-energy structures of Ca^2+^(H_2_O)_n_ with *n* = 10–18 taken from Lei and Pan (2010) as well as Wales and co-workers (González et al., 2005) optimized at the B3LYP-D3/6-311+G(d,p) level of theory are shown in Supplementary Figure S1. The structures taken from Lei and Pan as well as Wales and co-workers are described as *n*-Lei and *n*-Wales, respectively.

**TABLE 1:** The number of water molecules in the first (N_1_), second (N_2_), and third (N_3_) hydrated shells, and the number of hydrogen bonds (N_HB_) of the lowest-energy structures of Ca^2+^(H_2_O)_10–18_ clusters.

*n*	N_1_	N_2_	N_3_	N_HB_
10	6	4	0	8
11	6	5	0	10
12	6	6	0	12
13	7	6	0	12
14	8	6	0	12
15	7	7	1	15
16	7	8	1	20
17	8	9	0	23
18	8	10	0	25

From Figure 1, we can see that, for all the lowest-energy structures, there are no hydrogen bonds between the water molecules in the first hydration shell and Ca^2+^ prefers to stay inside the cluster, in agreement with *n*-Lei, *n*-Wales, and the results of molecular dynamics simulations (Egorov et al., 2003; González et al., 2005; Lei and Pan, 2010). The relatively intensive electronic field makes the water molecules more relaxed and more difficult to form hydrogen bonds (González et al., 2005). For *n* = 10, there are no hydrogen bonds between the water molecules in the second shell. While from *n* = 11, there are hydrogen bonds between the water molecules in the second hydration shell. Meanwhile, only Ca^2+^(H_2_O)_15_ and Ca^2+^(H_2_O)_16_ have a water molecule in the third hydration shell.

As shown in Table 1, in the beginning, the first hydration shell of the calcium ion is fully occupied with six water molecules, which is a distorted octahedral core. As the number of water molecules increases, N_1_ increases to eight. The transition of N_1_ from six to eight begins at about *n* = 12, which is corresponding to the experimental results of Bush et al. (2008). As the number of water molecules increases, N_1_ fluctuates between seven and eight. The number of water molecules in the second shell increases monotonously with the number of water molecules increasing. Like the number of water molecules in the second shell, the number of hydrogen bonds increases as the number of water molecules increases. Thus, in the range of *n* = 10–18, N_1_ and N_2_ are not exactly six and nine, respectively, which is not the same as the simulation of Lei and Pan (2010). There is a strong competition between the first and second hydration shell water molecules.

**FIGURE 2:**
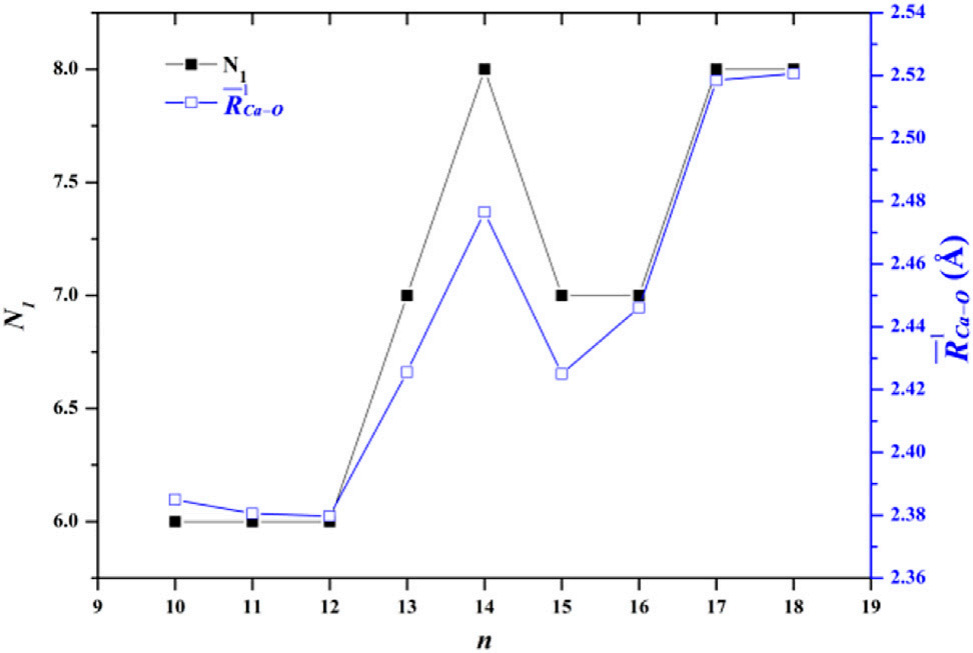
Comparison between the number of water molecules in the first hydration shell (N_1_) and the average adjacent Ca-O distance as a function of the number of water molecules.

The average adjacent O-O distance and the average Ca-O distance between Ca^2+^ and oxygen atoms of the water molecules in the first, second, and third hydration shells of the lowest-energy structures of Ca^2+^(H_2_O)_10–18_ clusters are shown in Table 2. As the number of water molecules increases, the average adjacent O-O distance decreases, meaning that as the cluster size increases, the average interaction between water molecules becomes stronger. The average distance between Ca^2+^ and O in the first hydration shell water molecules varies from 2.38 to 2.521 Å, which are consistent with the results of MD simulation (Schwenk et al., 2001). Meanwhile, the variation trends of the coordination number of the first hydration shell as well as the average distance between Ca^2+^ and O in the first hydration shell with the cluster size are the same, as shown in Figure 2. Thus, the average distance between Ca^2+^ and O in the water molecules of the first hydration shell increases as the number of first-shell water molecules increases, which have the same trend with the previous works (Bakó et al., 2002; Carl et al., 2007; Lei and Pan, 2010). The average distance between Ca^2+^ and O in the second hydration shell increases as the cluster size increases for *n* = 10–14, while it decreases as the cluster size increases for 14 < *n* ≤ 18. Thus, the distance between the first and second hydration shells decreases as the number of water molecules increases. Meanwhile, from *n* = 14, when the number of water molecules in the first hydration shell remains seven or eight, the number of water molecules in the second hydration shell still increases, and the water molecules in the second hydration shell generate hydrogen bonds. The geometric characteristic of Ca^2+^(H_2_O)_10–18_ clusters reveals that as the cluster size increases, the interaction between Ca^2+^ and water molecules decreases and the interaction between water molecules increases.

**TABLE 2:** Average adjacent O-O distance (${\bar{R}}_{O-O}$) and the average Ca-O distance between Ca^2+^ and oxygen atoms in the first (${\bar{R}}_{Ca-O}^{1}$), second (${\bar{R}}_{Ca-O}^{2}$), and third (${\bar{R}}_{Ca-O}^{3}$) shell water molecules of the lowest-energy structures of Ca^2+^(H_2_O)_10–18_ clusters.

*n*	${\bar{R}}_{O-O}Å$	${\bar{R}}_{Ca-O}^{1}Å$	${\bar{R}}_{Ca-O}^{2}Å$	${\bar{R}}_{Ca-O}^{3}Å$
10	2.818	2.385	4.257	—
11	2.825	2.381	4.275	—
12	2.839	2.380	4.281	—
13	2.852	2.426	4.350	—
14	2.822	2.477	4.528	—
15	2.843	2.425	4.411	6.149
16	2.791	2.446	4.169	5.005
17	2.775	2.518	4.083	—
18	2.781	2.521	4.106	—

### Relative Energy and Stability

The energy differences at the MP2/6-311++G(2d,2p) level of theory between the structures obtained from the CGA and *n*-Lei and *n*-Wales are shown in Table 3. As shown in Figure 1, Supplementary Figure S1, and Table 3, for Ca^2+^(H_2_O)_10_, the number of first hydration shell water molecules is six. The structure is different from 10-Lei and 10-Wales, while has the same number of hydrogen bonds with 10-Lei. Ca^2+^(H_2_O)_10_ has higher symmetry and lower total energy by 3.87 kJ/mol compared with 10-Lei and 10-Wales. Ca^2+^(H_2_O)_11_ is also different from 11-Lei and 11-Wales with lower energy. There are hydrogen bonds between the water molecules in the second shell. Ca^2+^(H_2_O)_12_ is the same with 12-Lei, which is 6.69 kJ/mol lower in energy than 12-Wales. For Ca^2+^(H_2_O)_13_, there are seven water molecules in the first hydration shell. Ca^2+^(H_2_O)_13_ is 2.10 kJ/mol higher in energy than 13-Lei and 7.31 kJ/mol lower in energy than 13-Wales, respectively. Ca^2+^(H_2_O)_14_ has eight water molecules in the first shell and is 1.87 kJ/mol higher in energy than 14-Lei as well as 0.12 kJ/mol higher in energy than 14-Wales. For Ca^2+^(H_2_O)_15_, the third-shell water molecule appears. Meanwhile, Ca^2+^(H_2_O)_15_ is 0.17 kJ/mol lower in energy than 15-Lei and 0.11 kJ/mol higher in energy than 15-Wales. Ca^2+^(H_2_O)_16_ is 0.33 kJ/mol lower in energy than 16-Lei, while is 3.57 kJ/mol higher in energy than 16-Wales. Ca^2+^(H_2_O)_17_ and Ca^2+^(H_2_O)_18_ only have two hydration shells, and several water molecules in the second shell hydrogen bonded to three water molecules in the first shell. Ca^2+^(H_2_O)_17_ and Ca^2+^(H_2_O)_18_ are both more stable than the corresponding ones taken from the work of Lei and Pan, and higher in energy than the ones taken from the work of Wales and co-workers.

**TABLE 3:** The energy differences (in units of kJ/mol) between the structures obtained from the CGA and *n*-Lei as well as *n*-Wales calculated at MP2/6–311++G(2d,2p)//B3LYP-D3/6–311+G(d, p) level of theory with thermal correction at different temperature.

*n*	0K			298K		
	GA	Lei	Wales	GA	Lei	Wales
10	0	3.87	3.87	0	3.41	5.40
11	0	1.70	8.77	0	2.24	12.91
12	0	0	6.69	0	0	9.25
13	0	−2.10	7.31	0	−5.67	7.31
14	0	−1.87	−0.12	0	−5.01	−0.22
15	0	0.17	−0.11	0	−5.90	−2.96
16	0	0.33	−3.57	0	8.93	7.28
17	0	2.83	−7.22	0	17.25	5.41
18	0	9.75	−2.22	0	27.04	14.26

The above-mentioned energy differences are all at 0 K, while the thermal effect could change the relative stability of clusters (Lei and Pan, 2010; Shi et al., 2018b). Thus, we also provide the energy difference at room temperature in Table 3. For *n* = 10–12, the structures obtained from the CGA are the most stable ones both at 0 K and room temperature. For *n* = 13, 13-Lei are the most stable ones both at 0 and 298 K. The structures obtained from the CGA are more stable than the 13-Wales. However, for *n* = 14 and 15, *n*-Lei and *n*-Wales become more stable than Ca^2+^(H_2_O)_n_ from the CGA at room temperature. For *n* = 16–18, the structures obtained from the CGA are more stable than the *n*-Lei, while more unstable than the *n*-Wales at 0 K. As the temperature arises to 298 K, the structures obtained from the CGA become the most stable structures. In general, as the temperature increases as well as the cluster size increases, the number of water molecules in the first hydration shell is more favored between seven and eight, which is opposite with the trend derived by Bai and co-workers using *ab initio* molecular dynamic simulation (Bai et al., 2013). Meanwhile, as shown in Table 3, such small energy differences indicate that the structures obtained from the CGA, *n*-Lei, and *n*-Wales are concomitant both at 0 K and at room temperature.

The sequential water binding energy (ΔE_seq._) of a Ca^2+^(H_2_O)_n_ cluster is defined as follows:

$$\Delta {\text{E}}_{\text{seq}\text{.}}=\text{E}\left({\text{H}}_{2}\text{O}\right)+\text{E}\left[{\text{Ca}}^{2+}{\left({\text{H}}_{2}\text{O}\right)}_{\text{n}-1}\right]-\text{E}\left[{\text{Ca}}^{2+}{\left({\text{H}}_{2}\text{O}\right)}_{\text{n}}\right].$$ (2)

As shown in Table 4, the simulated sequential water binding energy of the lowest-energy structures of Ca^2+^(H_2_O)_n_ clusters with *n* = 11–18 is almost overlapped in the error bar though slightly larger than the corresponding experimental results with the same cluster size (Peschke et al., 1998; Bruzzi and Stace, 2017).

**TABLE 4:** The sequential water binding energy (ΔE_seq._, in units of kJ/mol) and the average interaction energy (E_I_/N_1_, in units of kJ/mol) of the lowest-energy structures of Ca^2+^(H_2_O)_10–18_ clusters.

*n*	ΔE_seq_	E_exp_	E_I_/N_1_
10	—	—	−227.7
11	56.3	48 ± 7^a^ 55.7^b^	−235.3
12	57.0	44 ± 6.4^a^ 54.4^b^	−245.7
13	55.2	43 ± 2.4^a^ 51.9^b^	−221.0
14	49.1	46 ± 4.3^a^ 49.8^b^	−198.4
15	51.8	40 ± 5.9^a^	−234.4
16	46.7	38 ± 3.2^a^	−211.0
17	47.7	41 ± 5.9^a^	−179.4
18	51.5	48 ± 4.1^a^	−182.4

a Taken from Bruzzi and Stace, 2017.

b Taken from Peschke et al., 1998.

**FIGURE 3:**
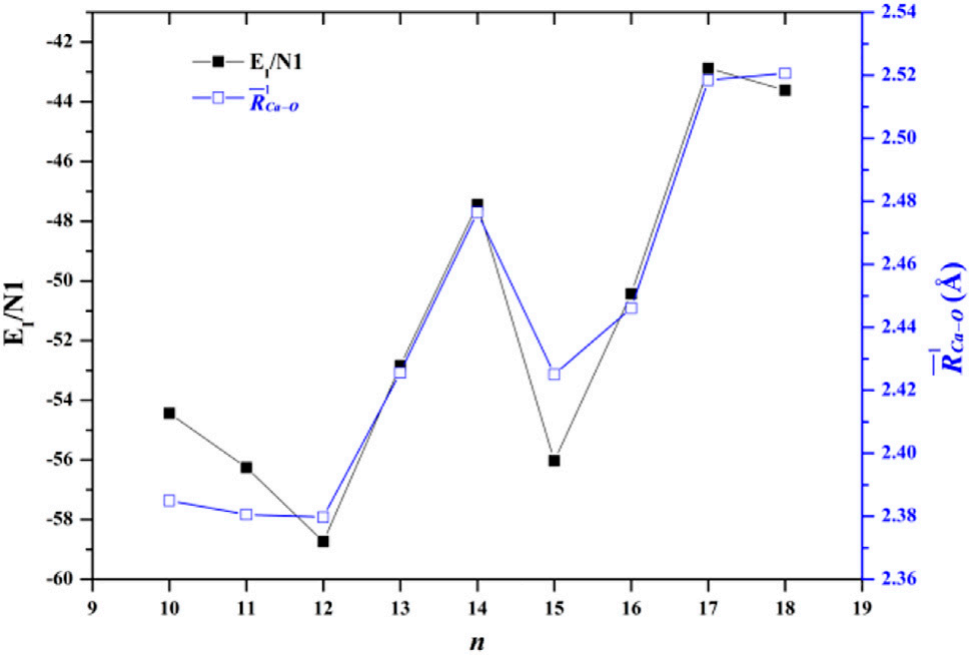
Comparison between the average interaction energy and the average adjacent Ca-O distance as a function of the number of water molecules.

Another significant energetic property of the Ca^2+^(H_2_O)_n_ cluster is the interaction energy between the calcium ion and water molecules. The average interaction energy between the calcium ion and adjacent water molecules of a Ca^2+^(H_2_O)_n_ cluster (E_I_/N_1_) is defined as follows:

$${\text{E}}_{\text{I}}/{\text{N}}_{1}=\left[\text{E}\left[{\text{Ca}}^{2+}{\left({\text{H}}_{2}\text{O}\right)}_{\text{n}}\right]-\text{E}\left({\text{Ca}}^{2+}\right)-\text{E}\left[{\left({\text{H}}_{2}\text{O}\right)}_{\text{n}}\right]\right]/{\text{N}}_{1},$$ (3)

where E[(H_2_O)_n_] is the energy of all the water molecules in the same geometry as in the cluster. The E_I_/N_1_ of the lowest-energy structures of Ca^2+^(H_2_O)_10–18_ clusters are listed in Table 4. Figure 3 also shows the comparison between the E_I_/N_1_ and the average adjacent Ca-O distances as a function of the number of water molecules. As the average distance between Ca^2+^ and oxygen atoms in the first-shell water molecules increases, the interaction between Ca^2+^ and water molecules decreases. Furthermore, the trends of the E_I_/N_1_ and the average adjacent Ca-O distances as a function of the number of water molecules are similar, indicating that the interaction between Ca^2+^ and water molecules mainly originates from Ca^2+^ and water molecules in the first-shell water molecules (Lei and Pan, 2010).

### Natural Bond Orbital

NBO analyses could give the information about the natural charge of every atom and charge transfer between different units and so on. Table 5 provides the average natural charge of the calcium ion and the oxygen atoms in the first, second, and third solvation shells. The average natural charge of Ca^2+^ becomes smaller as the number of first-shell water molecules increases. The average natural charge of the oxygen atoms in the first-shell water molecules is smaller than that in the second- and third-shell water molecules. Meanwhile, the average natural charge of the oxygen atoms in the second and third hydration shells remains almost unchanged. Therefore, the interaction between Ca^2+^ and water molecules mainly focuses on the interplay between the calcium ion and the first hydration shell (Lei and Pan, 2010).

**TABLE 5:** Average natural charge of the calcium ion δ(Ca^2+^) and the oxygen atoms in the first δ(O_1_), second δ(O_2_), and third δ(O_3_) solvation shells of the lowest-energy structures of Ca^2+^(H_2_O)_10–18_ clusters.

	δ(Ca^2+^)	δ(O_1_)	δ(O_2_)	δ(O_3_)
10	1.900	−1.047	−0.988	—
11	1.899	−1.050	−0.990	—
12	1.898	−1.055	−0.987	—
13	1.886	−1.038	−0.982	—
14	1.880	−1.023	−0.990	—
15	1.885	−1.040	−0.988	−0.979
16	1.883	−1.052	−1.009	−1.014
17	1.873	−1.053	−1.013	—
18	1.873	−1.056	−1.013	—

**FIGURE 4:**
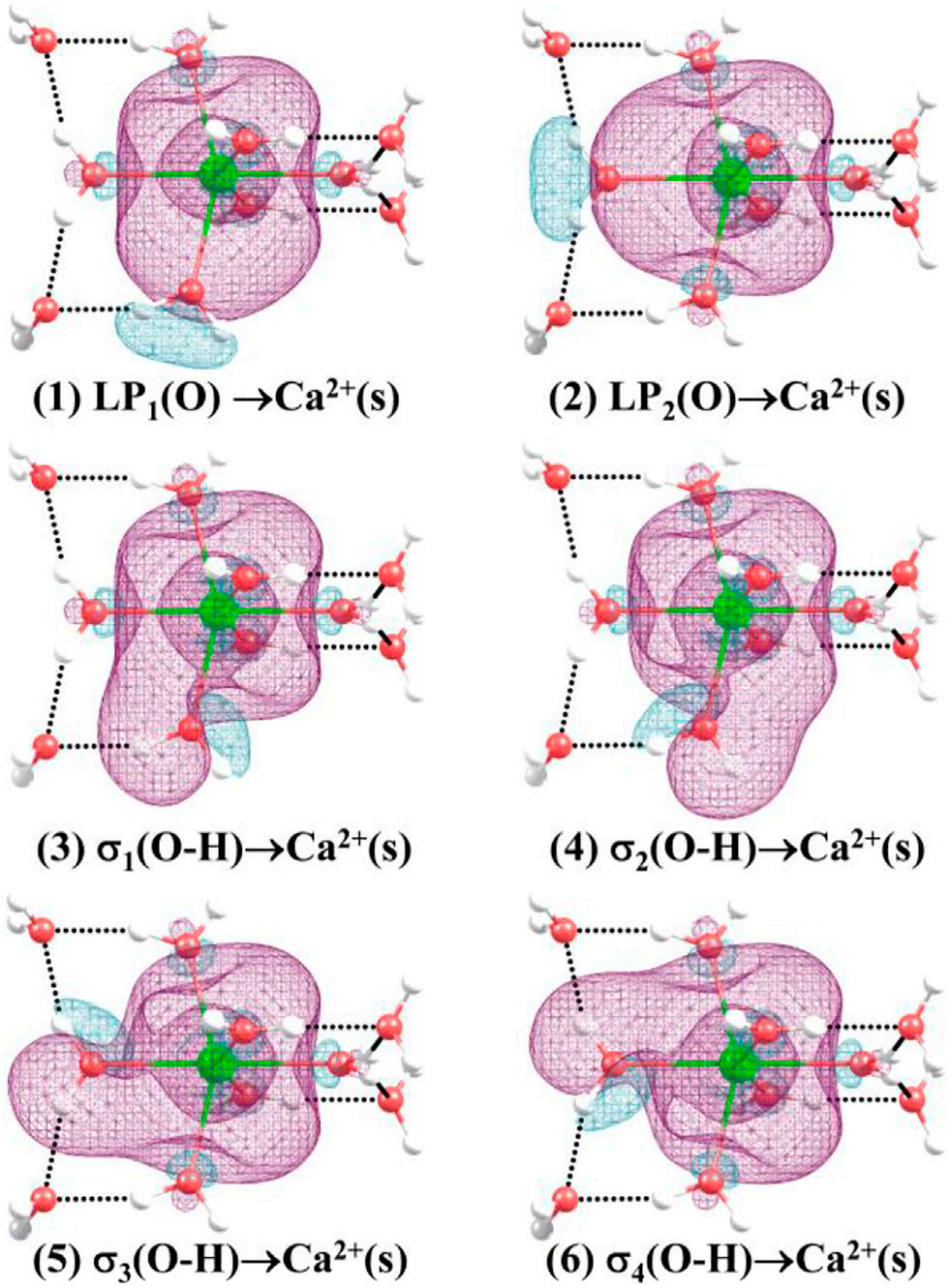
Three-dimensional schematics of the charge transfer from (1) to (2): the lone pair electron orbital of adjacent oxygen atoms, and (3)–(6): the bonding orbital of adjacent O-H to the empty orbital of Ca^2+^ for the lowest-energy structure of Ca^2+^(H_2_O)_10_.

Table 6 shows the second-order perturbation energy of the charge transfer between Ca^2+^ and water molecules, which results in the interaction between Ca^2+^ and adjacent water molecules. The charge transfer from the lone pair electron orbital of adjacent oxygen atoms and the bonding orbital of adjacent O-H to the empty orbital of Ca^2+^ occurs in all the lowest-energy structures of Ca^2+^(H_2_O)_10–18_ clusters. Figure 4 provides the schematic diagrams of these two kinds of charge transfer in the lowest-energy structure of Ca^2+^(H_2_O)_10_ clusters. Among them, the charge transfer from the lone pair electron orbital of adjacent oxygen atoms to the empty orbital of Ca^2+^ plays a leading role. Moreover, for the lowest-energy structures of Ca^2+^(H_2_O)_n_ clusters with *n* = 14, 17, and 18, the empty orbital of Ca^2+^ is not exactly empty. There is charge transfer from the empty orbital of Ca^2+^ to the Rydberg orbital of adjacent oxygen atoms, adjacent hydrogen atoms, and Ca^2+^ as well as to the antibonding orbital of adjacent O-H. Thus, the electron of Ca^2+^ becomes more diffused as the number of first-shell water molecules is eight.

**TABLE 6:** Average second-order perturbation energy (in units of kcal/mol) of several kinds of charge transfer of the lowest-energy structures of Ca^2+^(H_2_O)_10–18_ clusters.^a^

*n*	LP(O)-LP^*^(Ca)	BD(O-H)-LP^*^(Ca)	LP^*^(Ca)-RY^*^(O)	LP^*^(Ca)-RY^*^(H)	LP^*^(Ca)-BD^*^(O-H)	LP^*^(Ca)-RY^*^(Ca)
10	86.98	16.96	—	—	—	—
11	87.44	17.63	—	—	—	—
12	86.80	18.05	—	—	—	—
13	106.19	22.04	—	—	—	—
14	118.65	25.12	305.52	34.54	--	58.56
15	105.86	22.28	—	—	—	—
16	101.11	23.73	—	—	—	—
17	109.36	29.72	267.95	17.35	43.87	92.48
18	108.12	31.26	170.70	21.08	54.17	90.04

a LP and LP^*^ represent the lone pair electron orbital and the empty orbital, respectively. BD and BD^*^ represent the bonding orbital and antibonding orbital, respectively. RY^*^ is the Rydberg orbital.

## Conclusion

By means of CGA combined with DMol^3^ package, we search the potential energy surface of the hydrated calcium ion clusters Ca^2+^(H_2_O)_n_ with *n* = 10–18. The low-lying structures of Ca^2+^(H_2_O)_n_ clusters obtained from the CGA are re-optimized at the B3LYP-D3/6-311+G(d,p) level of theory. The lowest-energy structures of Ca^2+^(H_2_O)_10–18_ clusters reveal that Ca^2+^ prefers to locate at the center of the cluster. Meanwhile, the lowest-energy structures of Ca^2+^(H_2_O)_10–12_ clusters revalidate the conclusion that the coordination number of first-shell water molecules is six. The switch of the N_1_ from six to eight is with up to *n* = 12. As the cluster size rises to *n* = 18, the N_1_ fluctuates between seven and eight, indicating that there is a strong competition between the first and second hydration shell water molecules. The complexity of the lowest-energy structures of Ca^2+^(H_2_O)_10–18_ clusters increases as the cluster size increases since the number of the water molecules in the second shell and the total hydrogen bonds becomes more.

As the cluster size increases, the distance between Ca^2+^ and the adjacent water molecules increases while the average adjacent O-O distance decreases, implying that the interaction between Ca^2+^ and the adjacent water molecules becomes weaker and the interaction between water molecules becomes stronger. The interaction energy between Ca^2+^ and the water molecules, the natural charge, certifies that the interaction is mainly derived from the interaction between Ca^2+^ and the adjacent water molecules. Furthermore, the charge transfer from the lone pair electron orbital of adjacent oxygen atoms to the empty orbital of Ca^2+^ plays a leading role in the interaction between Ca^2+^ and water molecules.

## Data Availability

The original contributions presented in the study are included in the article/Supplementary Material; further inquiries can be directed to the corresponding author.
