# Supplementary material for: Ground-State Structures of Hydrated Calcium Ion Clusters From Comprehensive Genetic Algorithm Search
Source: Front Chem. 2021 Jun 30;9:637750. doi: 10.3389/fchem.2021.637750 (PMC8277924; doi:10.3389/fchem.2021.637750)
Supplement: Supplementary file 1 [file DataSheet1.docx]

**Ground-state structures of hydrated calcium ion clusters from comprehensive genetic algorithm search**

Ruili Shi,^1,2^ Zhi Zhao, ^1,2^ Xiaoming Huang,^3^ Pengju Wang,^2^ Yan Su,2[[1]](#footnote-1) Linwei Sai,^4^ Xiaoqing Liang,^5^ Haiyan Han,^1^ and Jijun Zhao^2^

*^1^ School of Mathematics and Physics, Hebei University of Engineering, Handan 056038, China*

*^2^ Key Laboratory of Materials Modification by Laser, Ion and Electron Beams, Dalian University of Technology, Ministry of Education, Dalian 116024, China*

*^3^ School of Ocean Science and Technology, Dalian University of Technology, Panjin Campus, Panjin 124221, China*

*^4^ Department of Mathematics and Physics, Hohai University, Changzhou 213022, China*

*^5^ School of Electronics and Information Engineering, Taizhou University, Taizhou 318000, China*


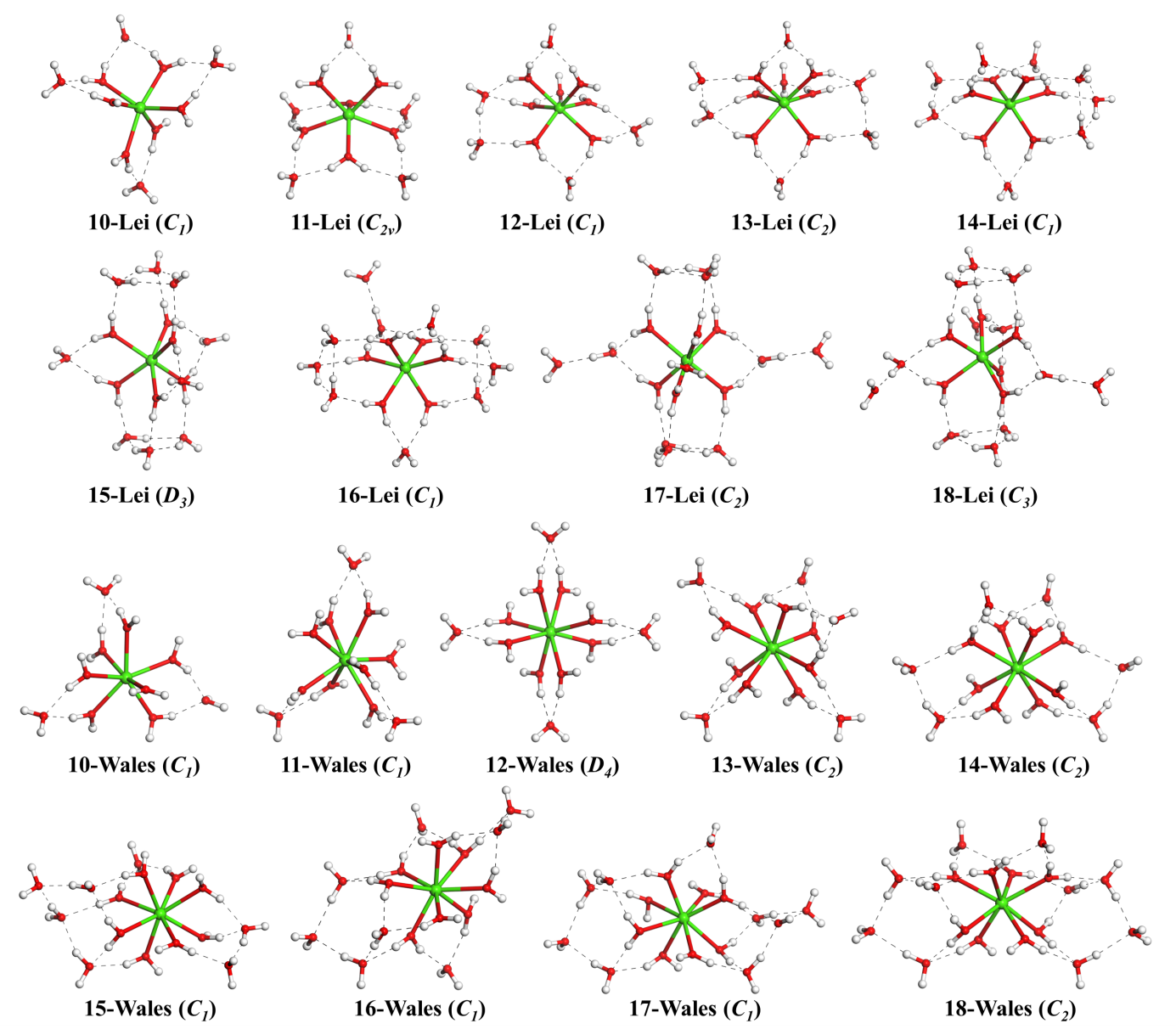


Figure S1. The lowest-energy structures of Ca^2+^(H_2_O)_n_ clusters with *n* = 10-18 from Ref. 1 and Ref. 2 optimized at the B3LYP-D3/6-311+G(d,p) level of theory. The symmetries in parentheses are the symmetries of clusters without hydrogen atoms. The green, red, and white balls represent Ca, O, and H atoms, respectively. The dashed black lines represent the hydrogen bonds.

Table S1. The differences of average adjacent O-O distances and average adjacent O-Ca distances of Ca^2+^(H_2_O)_10_ clusters between several methods and MP2/6-311++G(2d,2p) results. 10-GA, 10-Lei and 10-Wales are the lowest-energy structures obtained from CGA, Ref. 1 and Ref. 2, respectively.

| Method/basis set | 10-GA | | 10-Lei | | 10-Wales | |
| --- | --- | --- | --- | --- | --- | --- |
|  | χ_O-O_(%) | χ_Ca-O_(%) | χ_O-O_(%) | χ_Ca-O_(%) | χ_O-O_(%) | χ_Ca-O_(%) |
| MP2/6-311++G(2d,2p) | 0 | 0 | 0 | 0 | 0 | 0 |
| MP2/6-311++G(d,p) | 0.44 | 0.87 | 0.49 | -0.14 | 0.46 | -0.12 |
| MP2/6-311+G(d,p) | 0.42 | -0.12 | 0.48 | -0.12 | 0.43 | -0.08 |
| MP2/6-31+G(d,p) | 0.90 | 1.20 | 0.90 | 1.25 | 2.03 | 1.22 |
| BLYP/6-311+G(d,p) | 1.13 | 0.77 | 0.93 | 0.80 | 1.06 | 1.21 |
| B3LYP/6-311+G(d,p) | 0.43 | 0.08 | 0.41 | 0.10 | 0.37 | 0.37 |
| B3LYP-D3/6-311+G(d,p) | -0.08 | -0.28 | -0.16 | -0.26 | -0.11 | -0.28 |

χ_O-O_= [$\overline{R}_{O-O}$(method/basis set) − $\overline{R}_{O-O}$(MP2/6-311++G(2d,2p))]/$\overline{R}_{O-O}$(MP2/6-311++G(2d,2p))

χ_Ca-O_= [$\overline{R}_{Ca-O}$(method/basis set) − $\overline{R}_{Ca-O}$(MP2/6-311++G(2d,2p))]/$\overline{R}_{Ca-O}$(MP2/6-311++G(2d,2p))

Reference

1. X. Lei, and B. Pan, J. Phys. Chem. A **114**, 7595 (2010).

2. B. S. González, J. Hernández-Rojas, and D. J. Wales, Chem. Phys. Lett. **412**, 23 (2005).

1. Corresponding author: e-mail: su.yan@dlut.edu.cn [↑](#footnote-ref-1)
